# Evaluation of *DOK5 *as a susceptibility gene for type 2 diabetes and obesity in North Indian population

**DOI:** 10.1186/1471-2350-11-35

**Published:** 2010-02-27

**Authors:** Rubina Tabassum, Anubha Mahajan, Ganesh Chauhan, Om Prakash Dwivedi, Saurabh Ghosh, Nikhil Tandon, Dwaipayan Bharadwaj

**Affiliations:** 1 Functional Genomics Unit, Institute of Genomics and Integrative Biology (CSIR), Delhi- 110 007, India; 2 Human Genetics Unit, Indian Statistical Institute, Kolkata- 700 108, India; 3 Department of Endocrinology, All India Institute of Medical Sciences, New Delhi-110 029, India

## Abstract

**Background:**

Type 2 diabetes is a complex metabolic disorder with obesity being a major contributing factor in its development. Susceptibility loci for type 2 diabetes and obesity have been localized on different chromosomal regions by various genome-wide linkage scans. Of these chromosomal regions, 20q13 is one of the strongest linked regions for type 2 diabetes as well as obesity. On 20q13 lies *DOK5 *that seems to be a strong functional and positional candidate for type 2 diabetes and obesity because of its involvement in insulin signaling and immune responses. Hence, for the first time, we explored *DOK5 *as a potential type 2 diabetes and obesity susceptibility gene.

**Methods:**

We sequenced 43 subjects for polymorphisms in functionally relevant regions of *DOK5*. A total of 10 SNPs that included 5 that were identified by sequencing and 5 additional SNPs from NCBI Variation Database were genotyped in 2,115 participants comprising of 1,073 patients with type 2 diabetes and 1,042 controls of Indo-European ethnicity from North India.

**Results:**

We identified a novel variant in intron 7 referred to as DK176673. We found nominal association of three SNPs-rs6064099 (OR = 0.75, *P *= 0.019), rs873079 (OR = 0.76, *P *= 0.036) and DK176673 (OR = 1.55, *P *= 0.037) with type 2 diabetes among normal-weight subjects [BMI < 23 kg/m^2^]. The haplotype GGC harboring rs6068916, rs6064099 and rs873079 showed strong association with type 2 diabetes among normal-weight subjects (OR = 1.37, *P*/*P*_perm _= 5.8 × 10^-3^/0.037). Association analysis with obesity revealed that rs6064099 is associated with reduced susceptibility for obesity (OR = 0.48, *P *= 6.8 × 10^-3^). Also, haplotype GGC conferred increased susceptibility for obesity (OR = 1.27, *P*/*P*_perm _= 9.0 × 10^-3^/0.039). Also, rs6064099 was significantly associated with reduced BMI [median(IQR) = 24.0(20.7-27.1) vs 23.9(20.2-26.8) vs 21.8(19.2-24.7) for GG vs GC vs CC, *P *= 7.0 × 10^-3^].

**Conclusions:**

We identified *DOK5 *as a novel susceptibility gene for obesity and type 2 diabetes in North Indian subjects. Association of *DOK5 *variants both with obesity and type 2 diabetes suggests that these variants might modulate type 2 diabetes susceptibility through obesity.

## Background

Type 2 diabetes is a complex metabolic disorder characterized by impaired insulin secretion and action. Obesity is one of the major contributing factors in the development of type 2 diabetes. Though believed to be overlapping, the etiology of both type 2 diabetes and obesity are unclear. Gene identification is an important milestone in the understanding of disease pathophysiology, but has proven to be a difficult task for complex disorders such as type 2 diabetes. Multiple susceptibility loci on different chromosomal regions are believed to be involved in genetic etiology of type 2 diabetes.

Evidence for localization of susceptibility loci on different chromosomal regions for type 2 diabetes and obesity has been provided by various genome-wide linkage scans. Of these regions, 20q13 is one of the strongest candidate regions for type 2 diabetes which is documented to be linked to type 2 diabetes by more than 8 genome wide studies in different populations [1-9]. The same region has also been shown to be linked to obesity by various studies [10-12]. However, till date there has been no clear evidence for localization of type 2 diabetes and obesity susceptibility genes on this region. Hence, exploration of 20q13 through positional candidate approach may facilitate identification of susceptibility genes for type 2 diabetes and obesity on this region.

The region 20q13 harbors *DOK5 *that encodes Dok5 which belongs to the Downstream of Kinases (DOK) family containing tandem pleckstrin homology-phosphotyrosine binding (PH-PTB) domains at the N-terminal. Although the biological function of this docking protein is not very clear, Dok5 is shown to be one of the substrates in insulin signaling [13]. Dok5 contains a short C-terminus with potential sites for tyrosine phosphorylation that get phosphorylated in response to insulin and IGF1 [13]. Moreover, the highest expression of Dok5 has been detected in skeletal muscle which is the major tissue regulating metabolic homeostasis. Dok5 is also suggested to be involved in the regulation of immune response induced by T cells [14].

Because of its involvement in insulin signaling and immune responses which are the key modulating pathways in type 2 diabetes and obesity, *DOK5 *seems to be a convincing positional and functional candidate for type 2 diabetes and obesity. Therefore, here for the first time, we explored *DOK5 *as a potential type 2 diabetes and obesity susceptibility gene in North Indian population which has a high risk of developing type 2 diabetes.

## Methods

### Subjects' recruitment

A total of 2,115 unrelated subjects comprising of 1,073 patients with type 2 diabetes and 1,042 control subjects from North India belonging to Indo-European ethnicity were enrolled after obtaining written informed consent. Type 2 diabetic patients were recruited from Endocrinology clinic of All India Institute of Medical Sciences, New Delhi between the period of 2003 and 2008. Diagnosis of type 2 diabetes was done as per WHO criteria 2003 [15]. The control samples were collected by organizing 'Diabetes Awareness Camps' in the urban regions in and around Delhi. Subjects of ≥40 years of age without family history of diabetes who had glycated hemoglobin (HbA1c) level ≤6.0% and fasting glucose level <110 mg/dL were considered as controls. Detailed description of inclusion and exclusion criteria for cases and controls is provided previously [16]. Study was in accordance with the principles of the Helsinki Declaration and was approved by the Ethics Committees of the participating institutions.

### Anthropometric and biochemical characterization

All the recruited subjects underwent anthropometric and biochemical measurements. Height, weight, waist and hip circumferences, and blood pressure were measured following standard guidelines before drawing blood. Body mass index (BMI) and waist to hip ratio (WHR) were calculated from these measurements. Based on their BMI, the subjects were categorized into two groups according to the BMI cut-offs for Asian populations: normal-weight (BMI < 23 kg/m^2^) and over-weight/obese subjects (BMI ≥23 kg/m^2^) [17]. Venous blood samples were drawn after overnight fasting for biochemical measurements. Levels of glucose, HbA1c, insulin, C-peptide, total cholesterol, triglycerides (TG), high-density lipoprotein cholesterol (HDL-C), low-density lipoprotein cholesterol (LDL-C), urea, uric acid, creatinine and hsCRP were measured as described earlier [16,18].

### Screening and identification of polymorphisms

**Figure 1:**
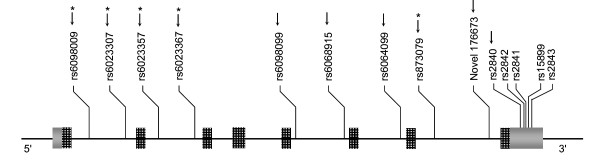
**Gene structure of *DOK5 *representing SNPs identified in Indian population and selected for association analysis**. SNPs marked with arrow were selected for association analysis. * indicates the SNPs selected from NCBI variation databases while other SNPs were identified by direct sequencing of the gene.

To identify novel variants if any, we sequenced approximately 5 kb region of *DOK5 *including all exons, exon-intron boundaries, putative promoter and UTRs in 43 samples of Indian Discovery panel [19]. Primers used were designed by Primer3 http://frodo.wi.mit.edu/primer3 that provides better primer design [20]. In the sequenced samples, we captured 9 SNPs including one novel variant in intron 7 (chromosomal position 53,266,929) that has never been reported earlier (referred here as DK176673) (Figure 1). From these, 5 SNPs (rs6098099, rs6068915, rs6064099, DK176673, rs2840) were selected for further genotyping. As SNPs in 3'UTR were very close, only one SNP (rs2840) was genotyped. Additionally, five more SNPs from NCBI Variation Database that includes rs6098009, rs6023307, rs6023357, rs6023367 and rs873079 were selected to cover the entire gene based on the selection criteria that includes functional significance, heterozygosity (MAF >0.05), information regarding tag SNPs and distance between the SNPs.

### Genotyping

Genotyping of these 10 SNPs was performed using GoldenGate assay on Illumina platform (Illumina Inc., San Diego, CA, USA). The genotyping data obtained was subjected to extensive quality control that includes genotype confidence score of 0.25, call frequency >0.9, GenTrans score >0.6, cluster separation score >0.4, MAF > 0.05 and Hardy-Weinberg equilibrium (HWE) in controls (*P *> 0.01). Of the total case-control samples, 90 DNA samples had genotype call for less than 90% of the SNPs and hence were excluded. A total of 147 samples (7%) were genotyped in duplicates and showed a consistency rate of 99.99% in genotype calls. SNP rs2840 failed in Illumina assay and was genotyped using MALDI-TOF mass spectrometry (Sequenom, San Diego, CA, USA).

### Statistical Analysis

Deviation from HWE at each locus was tested both among cases and controls separately using χ^2 ^analyses. Association of genotypes with type 2 diabetes and obesity was assessed by logistic regression. Association analyses of genotypes with type 2 diabetes were also performed after stratification of cases and controls into normal-weight and over-weight/obese subjects. The analyses were adjusted for age, sex and BMI as appropriate. Odds ratios (ORs) are presented with respect to the minor alleles. Bonferroni correction was applied to correct for multiple comparisons and a *P *value <0.008 was considered significant after correction. The uncorrected *P *values are provided in the text. Association between genotypes and quantitative traits was determined using Kruskal-Wallis test only in control subjects. Haplotype analysis was carried out at 10,000 permutations using Haploview 4.0 [21]. Statistical power was determined using PS power and sample size program [22]. Our sample provided power of 69%-97% to detect association with ORs of 1.3-1.5 assuming MAF of 0.20 at level of significance of 0.05. The statistical analyses were performed using SPSS version 17.0 (SPSS, Chicago, IL, USA) and PLINK v. 1.05 http://pngu.mgh.harvard.edu/~purcell/plink[23].

## Results

### DOK5 polymorphisms

We identified a total of 9 SNPs after sequencing the functionally significant regions of *DOK5 *including one novel SNP in intron 7 (referred as DK176673) and 8 reported SNPs-rs6098099 (Intron 5), rs6068915 (Intron 5), rs6064099 (Intron 6), rs2840 (5'UTR), rs2842 (5'UTR), rs2841 (5'UTR), rs15899 (5'UTR) and rs2843 (5'UTR) (Figure 1). As the 5 SNPs identified in 5'UTR region were very close to each other, only one (rs2840) was selected for further genotyping. Hence, of the 9 identified SNPs, 5 SNPs- rs6098099, rs6068915, rs6064099, DK176673, rs2840 were selected for further genotyping. Additionally, five more SNPs from NCBI Variation database that includes rs6098009 (Intron 1), rs6023307 (Intron 1), rs6023357 (Intron 2), rs6023367 (Intron 2) and rs873079 (Intron 7) were also selected for the complete coverage of gene region.

### DOK5 polymorphisms and type 2 diabetes

A total of 10 SNPs were genotyped in the study population of 2,115 participants comprising of 1,073 patients with type 2 diabetes and 1,042 controls of Indo-European ethnicity from North India. The clinical characteristics of the study population are provided in Table 1. All the SNPs were in accordance with HWE (all *P *> 0.01) both among cases and controls, except for rs2840 that was eliminated from further analysis. SNPs-rs6098009, rs6023307 and rs6098099 were found to be rare variants (MAF < 0.05) and were consequently excluded under the paradigm of a CVCD model. None of the *DOK5 *variants analyzed here showed association with type 2 diabetes (Table 2).

**Table 1:** Anthropometric and clinical characteristics of the study population

Characteristics	Type 2 diabetic patients	Control subjects
N (Men/Women)	1019 (592/427)	1006 (606/400)
Age (years)	53 (45-62)	50 (44-60)
BMI (Kg/m^2^)		
Men	23.8 (22.0-26.0)	23.1 (20.1-25.7)
Women	26.7 (24.2-29.2)	25.0 (21.1-28.5)
WHR		
Men	1.0 (0.97-1.03)	0.97 (0.92-1.0)
Women	1.0 (0.97-1.03)	0.86 (0.82-0.92)
Systolic BP (mmHg)	130 (130-140)	120 (112-132)
Diastolic BP (mmHg)	80 (78-90)	80 (70-88)
HbA1c (%)	7.8 (6.5-9.4)	5.2 (4.9-5.6)
Fasting Glucose (mmoles/L)	7.9 (6.4-10.3)	4.9 (4.5-5.3)
Fasting Insulin (μU/mL)	13.8 (7.0-27.8)	5.4 (2.9-9.6)
HOMA-IR	5.2 (2.3-9.6)	1.2 (0.6-2.0)
C-peptide (ng/mL)	2.7 (1.7-4.1)	1.6 (1.1-2.2)
hsCRP (mg/L)	2.2 (0.9-4.7)	1.3 (0.6-3.0)
Total cholesterol (mg/dL)	163 (137-195)	170 (146-199)
LDL-C (mg/dL)	100 (77-130)	108 (90-132)
HDL-C (mg/dL)	40 (35-48)	41 (34-50)
TG (mg/dL)	138 (100-198)	117 (86-161)
Urea (mg/dL)	26 (20-33)	24 (19-29)
Uric acid (mg/dL)	4.9 (3.9-6.0)	4.9 (4.0-5.7)
Creatinine (mg/dL)	0.84 (0.67-1.07)	0.75 (0.66-0.88)

Data are median values with interquartile ranges in parentheses.

**Table 2:** Association of *DOK5 *SNPs with type 2 diabetes in North Indian population

SNP	Entire Sample set			Normal-weight subjects			Over-weight/obese subjects		
	
	Case: Control (1019: 1006)	OR (95%CI) *P *value	OR_**adj **_(95%CI) *P*_**adj **_value	Case: Control (295: 436)	OR (95%CI) *P *value	OR_**adj **_(95%CI) *P*_**adj **_value	Case: Control (691: 561)	OR (95%CI) *P *value	OR_**adj **_(95%CI) *P*_**adj **_value
rs6023357 (A/C)	814: 822 196: 165 7: 16	1.06 (0.86-1.30) 0.597	1.10 (0.90-1.36) 0.355	236: 351 56: 77 1: 6	0.96 (0.68-1.36) 0.825	0.95 (0.65-1.39) 0.799	548: 465 137: 87 6: 9	1.17 (0.90-1.52) 0.246	1.16 (0.89-1.50) 0.276
rs6023367 (G/A)	794: 792 218: 202 7: 11	1.03 (0.84-1.25) 0.810	1.06 (0.86-1.29) 0.606	223: 341 70: 92 2: 3	1.14 (0.82-1.60) 0.432	1.19 (0.83-1.71) 0.335	548: 446 138: 107 5: 8	0.97 (0.75-1.26) 0.838	0.97 (0.75-1.26) 0.829
rs6068915 (C/T)	772: 759 229: 217 17: 28	0.95 (0.79-1.13) 0.533	0.95 (0.79-1.13) 0.562	226: 324 63: 98 6: 14	0.87 (0.65-1.17) 0.361	0.91 (0.66-1.24) 0.538	525: 430 154: 116 11: 14	0.99 (0.79-1.25) 0.940	0.97 (0.77-1.23) 0.830
rs6064099 (G/C)	589: 576 366: 369 64: 60	1.00 (0.86-1.15) 0.948	1.00 (0.86-1.16) 0.990	185: 239 95: 161 15: 36	0.75 (0.59-0.95) **0.019**	0.78 (0.60-1.01) 0.063	385: 331 259: 207 47: 23	1.18 (0.98-1.42) 0.080	1.18 (0.98-1.43) 0.080
rs873079 (G/A)	607: 591 356: 364 56: 50	0.99 (0.86-1.15) 0.927	0.99 (0.85-1.15) 0.861	191: 248 91: 162 13: 26	0.76 (0.59-0.98) **0.036**	0.78 (0.59-1.02) 0.065	397: 337 253: 201 41: 23	1.14 (0.94-1.37) 0.186	1.14 (0.94-1.38) 0.176
DK176673 (T/A)	847: 807 107: 110 6: 1	1.02 (0.78-1.33) 0.900	1.04 (0.79-1.37) 0.791	236: 359 38: 43 5: 1	1.55 (1.03-2.34) **0.037**	1.58 (1.01-2.46) **0.043**	584: 441 63: 66 1: 0	0.75 (0.52-1.07) 0.116	0.76 (0.53-1.09) 0.132

Data are presented as genotype counts. OR (95% CI) stands for odds ratio with 95% confidence interval assuming additive model. OR_adj _and *P*_adj _were calculated by logistic regression analysis after adjustments for age, sex and BMI.

Obesity is a well known risk factor of type 2 diabetes and influence of BMI on the risk of development of type 2 diabetes has been consistently shown in number of studies [24]. Recently, we also showed variability in the risk of type 2 diabetes among individuals in different BMI strata [16,25]. This implies that the etiology of type 2 diabetes might be different in normal-weight individuals and overweight/obese individuals. Therefore, we segregated the subjects into two groups based on BMI: Normal-weight (BMI < 23 kg/m^2^) and overweight/obese (BMI ≥ 23 kg/m^2^). Type 2 diabetes patients among normal-weight and over-weight/obese groups had median age of 55 years with median BMI of 21.3 kg/m^2 ^and 53 years of median age with median BMI of 26.5 kg/m^2 ^respectively. The control subjects among normal-weight and over-weight/obese groups had median age of 50 years with median BMI of 20.0 kg/m^2 ^and 50 years of median age with median BMI of 26.4 kg/m^2 ^respectively. Then, we compared the allelic and genotypic distributions among normal-weight cases and controls; and overweight/obese cases and controls (Table 2). We observed nominal association of rs6064099 and rs873079 among normal-weight subjects [OR = 0.75 (*P *= 0.019) and 0.76 (*P *= 0.036)]. The association did not remain significant after adjusting for age, sex and BMI. Also, DK176673 was found to confer susceptibility to type 2 diabetes (OR = 1.55, *P *= 0.037) that was retained after adjustments for covariates (*P *= 0.043) but did not after applying multiple testing correction. Among over-weight/obese subjects, none of the SNPs showed significant effect on type 2 diabetes susceptibility.

SNPs rs6064099 and rs873079 were in strong LD (D' = 0.98 and r^2 ^= 0.91). Among normal-weight individuals, haplotype GGC harboring major alleles of rs6068916, rs6064099 and rs873079 was more frequent among type 2 diabetes patients (66.9%) as compared to control subjects (59.8%). The GGC haplotype was found to confer increased susceptibility for type 2 diabetes (OR = 1.37, *P*/*P*_perm _= 5.8 × 10^-3^/0.037) among normal-weight individuals (Table 3). Another haplotype CTT encompassing rs6064099, rs873079 and DK176673 showed reduction in type 2 diabetes susceptibility among normal-weight subjects, however did not remain significant after permutation analyses (haplotype frequencies of 19.5% and 24.1% among type 2 diabetes patients and controls respectively; OR = 0.76, *P*/*P*_perm _= 0.038/0.242).

**Table 3:** Association of haplotype GGC with type 2 diabetes and obesity in North Indian population

Subjects	Haplotype Frequency	OR (95%CI)	*P *value	*P*_**perm **_value
Type 2 diabetic patients	66.9	1.37 (1.10-1.70)	0.0058	0.037
Non-diabetic controls	59.8			

NW controls	59.7	1.27 (1.06-1.53)	0.009	0.039
OW controls	65.4			

Haplotype frequencies are provided in percentages. NW stands for normal-weight subjects whereas OW stands for over-weight/obese subjects; OR (95% CI) stands for odds ratio with 95% confidence interval. *P*_perm _represents the *P *values obtained after performing 10,000 permutations.

### DOK5 polymorphisms and obesity

Association of *DOK5 *variants with obesity was assessed by considering BMI both as continuous and discrete trait (≥23 kg/m^2^) among control subjects. SNP rs6064099 was significantly associated with reduced BMI [median(IQR) = 24.0(20.7-27.1) vs 23.9(20.2-26.8) vs 21.8(19.2-24.7) for GG vs GC vs CC, *P *= 7.0 × 10^-3^]. Considering BMI as a discrete trait, we observed significant association of rs6064099 with obesity (OR = 0.48, *P *= 6.8 × 10^-3^) that remained significant after adjusting for age and sex (*P *= 9.8 × 10^-3^). We also found significant association of GGC haplotype with obesity. GGC haplotype was over-represented among over-weight/obese subjects (65.4%) compared to normal-weight subjects (59.7%), conferring risk for obesity with OR of 1.27 [*P *= 9.0 × 10^-3^/*P*_perm _= 0.039].

### DOK5 polymorphisms and quantitative clinical traits

Further, we investigated association of *DOK5 *SNPs with quantitative traits related to type 2 diabetes including fasting glucose, HbA_1c_, insulin, C-peptide, hsCRP, total cholesterol, HDL, LDL, triglyceride, creatinine, urea and uric acid. For this, the clinical variables of only control subjects were compared across the genotypes of the SNPs as the disease status or treatment regime in patients might affect the estimation of these parameters. However, none of the SNPs was found to be significantly associated with the clinical traits investigated here (all *P *> 0.05).

## Discussion

Indian population represents the highest risk group for type 2 diabetes and related metabolic traits including obesity and cardiovascular diseases. A number of association studies have been performed in Indian population to evaluate the role of functional candidate genes, most of which involves replication of associations in other populations. Moreover, these efforts have not yielded any true susceptibility gene that causes type 2 diabetes in this high risk group. With the advent of genome-wide association (GWA) studies, there has been a sudden increase in the number of confirmed loci for type 2 diabetes [26]. However, these identified loci contribute only to a small proportion of expected number of involved genes [27]. Hence, along with the model free approach of GWA studies, positional candidate approach targeting genes with plausible functional relevance can substantially contribute to a better understanding of the complex disorders. Therefore, here, we investigated a potential positional and functional candidate gene, *DOK5 *on chromosomal region 20q13, to identify novel susceptibility gene for type 2 diabetes and obesity.

Association analysis of *DOK5 *SNPs revealed significant association of its variants with type 2 diabetes among normal-weight subjects. SNPs rs6064099 in intron 6 and rs873079 in intron 7 were found to reduce the susceptibility to type 2 diabetes among normal-weight individuals. Also, the novel SNP DK176673 was found to confer susceptibility to type 2 diabetes. Normal-weight individuals harboring haplotype GGC of major alleles for SNPs rs6068915, rs6064099 and rs873079 were found to be more susceptible to type 2 diabetes. Hence, our data suggests that *DOK5 *might play a significant role in modulating the susceptibility of type 2 diabetes among normal-weight subjects in North Indian population. Our study further reinforces our earlier observations that etiologic mechanisms of pathophysiology of type 2 diabetes depend on BMI [11,24].

Consistent with earlier observations of linkage of 20q13 with obesity, our study also provides added evidence for the localization of susceptibility gene for obesity in this region. We found strong association of rs6064099 with protection against obesity. Also, GGC haplotype was found to confer increased susceptibility to obesity. Association of *DOK5 *variants with obesity again suggests that these variants may modulate the susceptibility to type 2 diabetes through obesity.

It is interesting to note the GGC haplotype region harboring three SNPs rs6068916, rs6064099 and rs873079, encompasses exons 6 and 7 of DOK5 gene. In case if these associated SNPs are not true causal variants, there might be a causal or functionally relevant variant in LD with the associated SNPs influencing the risk of type 2 diabetes and obesity. Though we attempted to identify all the polymorphisms in exonic regions, there might be possibility of less common variants with higher relative risks in these regions that could not be captured in our sequenced samples. Hence, further evaluation of SNPs in *DOK5 *in a larger population might provide true susceptibility variant in *DOK5 *gene.

We would like to mention here that production of false positives in association studies due to population stratification and multiple comparisons might be plausible. With this in mind, our case and control subjects were recruited from a homogenous cluster in urban region of North India in accordance to a report of genetic landscape of the people of India [28]. From the clustering pattern, it was suggested that if cases and controls are both drawn from the same cluster, the effects of population stratification in disease association studies may be small. Another study analyzing Indian genetic variation and diversity also suggested that the effects of population heterogeneity on the production of false positives in association studies might be smaller in Indians than might be expected for such a geographically and linguistically diverse subset of the human population [29].

## Conclusions

In conclusion, we identified *DOK5 *as a novel gene modulating the susceptibility of obesity and diabetes in North Indian population of Indo-European ethnicity. However, replication analyses of the variants showing association in this study are warranted to validate the findings. Moreover, other genetic variants in the gene might also play role in influencing the risk of type 2 diabetes. Therefore, future genetic and functional studies evaluating the association of genetic variants of *DOK5 *and deciphering their physiological effect and mechanisms are needed to further ascertain its role in the manifestation of type 2 diabetes.

## List of Abbreviations

DOK5: Docking Protein 5; IGF1: Insulin like Growth Factor 1; PH-PTB: pleckstrin homology-phosphotyrosine binding domain; CVCD: Common Variant Common Disease; MAF: Minor Allele Frequency

## Competing interests

The authors declare that they have no competing interests.

## Authors' contributions

RT and AM designed, processed, interpreted the data and wrote the manuscript. GC and OPD contributed in work design and manuscript writing. SG contributed in the statistical analysis of the data. DB and NT conceived and supervised the study and have contributed by critical evaluation of the study and improving the manuscript. All the authors read and approved the final manuscript.

## Pre-publication history

The pre-publication history for this paper can be accessed here:

http://www.biomedcentral.com/1471-2350/11/35/prepub
